# Genome mining of *Streptomyces scabrisporus* NF3 reveals symbiotic features including genes related to plant interactions

**DOI:** 10.1371/journal.pone.0192618

**Published:** 2018-02-15

**Authors:** Corina Diana Ceapă, Melissa Vázquez-Hernández, Stefany Daniela Rodríguez-Luna, Angélica Patricia Cruz Vázquez, Verónica Jiménez Suárez, Romina Rodríguez-Sanoja, Elena R. Alvarez-Buylla, Sergio Sánchez

**Affiliations:** 1 Departmento de Biología Molecular y Biotecnología, Instituto de Investigaciones Biomédicas, Universidad Nacional Autónoma de México (UNAM), Ciudad de México, México; 2 Laboratorio de Genética Molecular, Epigenética, Desarrollo y Evolución de Plantas, Instituto de Ecología, Universidad Nacional Autónoma de México (UNAM), Ciudad de México, México; 3 Instituto Tecnológico de Tuxtla Gutiérrez,Tuxtla, Gutiérrez, Chiapas, México; Universite Paris-Sud, FRANCE

## Abstract

Endophytic bacteria are wide-spread and associated with plant physiological benefits, yet their genomes and secondary metabolites remain largely unidentified. In this study, we explored the genome of the endophyte *Streptomyces scabrisporus* NF3 for discovery of potential novel molecules as well as genes and metabolites involved in host interactions. The complete genomes of seven *Streptomyces* and three other more distantly related bacteria were used to define the functional landscape of this unique microbe. The *S*. *scabrisporus* NF3 genome is larger than the average *Streptomyces* genome and not structured for an obligate endosymbiotic lifestyle; this and the fact that can grow in R2YE media implies that it could include a soil-living stage. The genome displays an enrichment of genes associated with amino acid production, protein secretion, secondary metabolite and antioxidants production and xenobiotic degradation, indicating that *S*. *scabrisporus* NF3 could contribute to the metabolic enrichment of soil microbial communities and of its hosts. Importantly, besides its metabolic advantages, the genome showed evidence for differential functional specificity and diversification of plant interaction molecules, including genes for the production of plant hormones, stress resistance molecules, chitinases, antibiotics and siderophores. Given the diversity of *S*. *scabrisporus* mechanisms for host upkeep, we propose that these strategies were necessary for its adaptation to plant hosts and to face changes in environmental conditions.

## Introduction

The symbiotic relationships between microorganisms and their hosts have come under scrutiny, as beneficial active molecules are likely to be expressed during this type of interactions. In endophytic relationships, microorganisms gain protection from the environment and easy access to nutrients, in return providing benefits to their host. These include the transformation of proteins into forms digestible by the host[1,2], synthesis of essential vitamins or amino acids[3,4], priming of the host immune system[2,5], xenobiotic degradation[6–8], protection against pathogens[9–11] and overall fitness to the plant[12–14]. It is envisioned that soon, plant growth-promoting bacteria will begin to replace the use of chemicals in agriculture, horticulture, silviculture, and environmental cleanup strategies[15–18]. The genus *Streptomyces* exhibits a remarkable potential to produce secondary metabolites and exposes diverse and unique functional capacities as well as biological activities. The advent of ~omics technologies and the ability to analyze the resulting data extended our ability to uncover new molecules for medical and industrial use and to understand their mechanisms[19–20].

The genomes of terrestrial *Streptomyces*, which are large in comparison to those of other bacteria, indicate that these organisms possess diverse carbon, nitrogen, and iron uptake systems, multiple genes for developmental regulation, and a remarkable number of gene clusters to produce biologically active small molecules[21–23]. Ample evidence suggests that *Streptomyces* are quantitatively and qualitatively important in the rhizospheres of plants, where they may influence plant growth and protect plant roots against invasion by root pathogens[24–26]. Also, a number of *Streptomyces* have an endophytic lifestyle, living within a plant for at least part of its life cycle without causing apparent disease[27–29]. Endophytic *Streptomyces* spp. are documented to promote plant growth[25,30], enable nutrient procurement[31], phytohormone synthesis[32–34], antibiosis and local competition against pathogens[31,35], and induction of systemic defense responses[24,29,36].

*Streptomyces scabrisporus* NF3 is an endophyte belonging to the Actinobacteria isolated from the tree *Amphipterygium adstringens*, endemic of Mexico (Rodriguez-Peña et al., in preparation). Samples of the tree were collected in the town of Barranca Honda in Yautepec, Morelos. The extract of this tree is used in traditional native medicine for the treatment of fever, fresh wounds, hypercholesterolemia, cholelithiasis, gastritis, gastric ulcers, gastrointestinal cancer and other inflammatory conditions[37–40].

While few strains of the species have been isolated and characterized in the past (mostly from soils) [35,41–43], only one strain of *S*. *scabrisporus* was sequenced and published so far: DSM 41855, isolated from soil from Japan. The genome of this strain is composed of 199 contigs, making it very difficult to use it in comparative analyses[44]. The few genomes sequenced of *S*. *scabrisporus* may be linked to the difficulty in obtaining cultured isolates, due to its slow growth rate in laboratory conditions and to its occurrence as an endophyte. *S*. *scabrisporus* has a great potential to produce diverse antimicrobial compounds against a wide range of microorganisms, among which, characterized so far are okilactomycin[43], hitachimycin[41,44] and their derivates. Antitumoral activity for alborixin has also been reported[45].

Here were report the exploration of the draft genome sequence of *S*. *scabrisporus* NF3 (SS NF3), and its comparison to ten complete genomes of biologically relevant strains, among which seven are taxonomical relatives from the family *Streptomycetaceae*: *S*. *venezuelae* ATCC 15439 (SV), *S*. *coelicolor* A3 (SC), *Kitasatospora setae* KM-6054 *(K*. *setae*, *KS)*, *S*. *avermitilis* MA-4680 (SA), *S*. *hygroscopicus* subsp. *limoneus* KCTC 1717 (SH), *Streptomyces* sp. TLI_053 (ST), *S*. *griseus* subsp. *griseus* NBRC 13350 (SG), and three more distantly related: *Bacillus anthracis* Sterne (BA) (mammalian intestinal pathogen), *Corynebacterium pseudotuberculosis* C231 (mammalian blood pathogen) (CP) and *Bifidobacterium breve* DSM 20213 (mammalian intestinal commensal) (BB). Several comparisons were made including the sequences of the incomplete *S*. *scabrisporus* DSM 41855 (SS DSM 41855) (S1G Table). The genomes were purposely selected not to be known endophytes, with the intention to detect an increase in gene abundance for plant interaction related genes in the genome of interest.

The comparison revealed an enrichment for genes associated with amino acid production and utilization, protein secretion, secondary metabolite and antioxidant production, and xenobiotic degradation, potentially indicating that SS contributes to the metabolic enrichment of its hosts. Exploration in the genome of SS NF3 revealed the presence of a large assortment of genes coding for the production of potential host interaction molecules like plant hormones, stress resistance molecules, chitinases, antibiotics, antibiotic resistance and siderophores. Among these genes, various numerous encode for prospective production of novel molecules.

## Results and discussion

### Genome assembly and gene prediction

**Fig 1:**
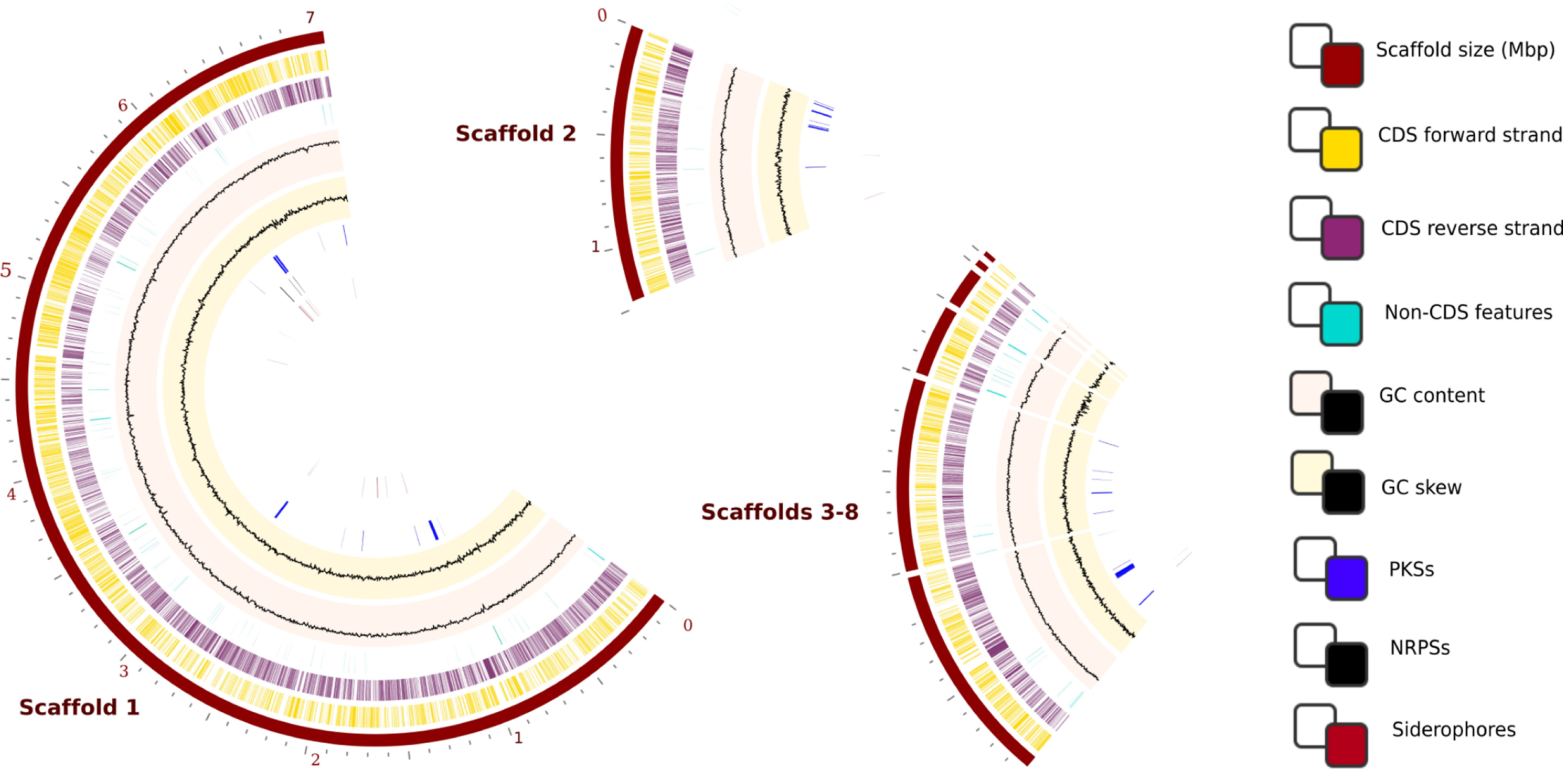
Genome characteristics for *S*. *scabrisporus* NF3. The circles refer to (from outside to inside): grey—Position label (Mbp): scaffold size in kbp; dark blue–contigs: organization of the scaffolds from the largest to the shortest; green–coding sequences on the forward strand (CDS FWD); purple–coding sequences on the reverse strand (CDS REV); blue–non-CDS features: location of tRNA, rRNA and pseudogenes; light purple–GC content; light orange–GC skew; dark green–location of genes encoding for polyketides; orange–location of genes encoding for nonribosomal peptide synthetases.

The genome sequence of SS NF3 was previously reported [28]. The genome is linear and has a 71.61% of GC content (Fig 1), falling into the normal range for a streptomycete and a size of 10.95 Mbp, falling in the top 5% of known *Streptomyces* genomes (S1 Fig). The gene prediction and annotation were made with two platforms, Prokaryotic Dynamic Programming Genefinding Algorithm (PRODIGAL) and the Rapid Annotations using Subsystems Technology (RAST) (S1A and S1B Table, respectively) and complemented with manual annotations based on domain and motif searches (S1A and S1C Table). Further analysis of the annotated genome was performed with Pathosystems Resource Integration Center (PATRIC) (Fig 1, S1A Table). The genome is predicted to contain 10,492 coding sequences (CDSs), 156 repeat regions, 60 tRNAs and 12 rRNAs, 5,691 predicted genes, 4,729 genes coding for hypothetical proteins and 382 pseudogenes (Fig 1). The origin of replication *oriC* is located after the *dnaA* gene (PEG.3184) at position 333,394 of scaffold 1, with the *oriC* repeats spanning over a 561 bp region. Table 1 shows the genome characteristics, as well as information about the genomes used for comparison.

**Fig 2:**
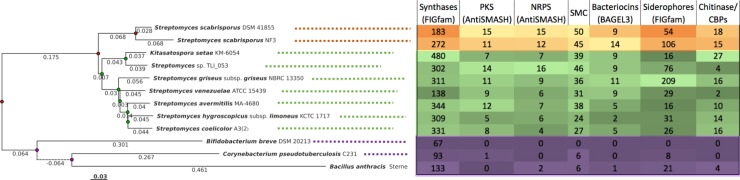
Phylogenetic tree of analyzed genomes. Phylogenetic trees were based on 4 concatenated proteins (16S rRNA, gyrB, rpoB, recG) conserved in all compared genomes using Kalign [46]. The tree was generated from a multiple sequence alignment using a Neighbor Joining algorithm. Bar shows substitution per nucleotide. The right side of the figure contains information about the abundance of genes of various functionalities discussed in the following paragraphs (synthases, siderophores and chitinases/chitin binding proteins (CBP)–computed using FIGfams, polyketide synthases (PKS), non-ribosomal peptide synthetases (NRPS) and total number of secondary metabolites clusters (SMC)–computed using antiSMASH, and bacteriocins–analyzed using BAGEL3).

Four highly conserved sequences (16S rRNA, gyrB, rpoB, recG) were used to construct a Neighbor Joining (NJ) phylogenetic tree of all compared genomes (Fig 2), which represents the evolutionary relatedness of the strains. As expected, an outgroup was formed with sequences of BA, CP and BB, while SS strains appear highly related. The position of KS between the other *Streptomyces* species was expected since this strain also belongs to the Streptomycetaceae family.

**Table 1:** General genome characteristics.

NCBI Taxon ID	Genome Name	Genome Status	Culture Collection	BioProject Accession	Assembly Accession	GenBank Accessions	Contigs	Genome Length	GC Content	PATRIC CDS	RefSeq CDS	Cell Shape	Sporul-ation	Oxygen Requirement	Habitat
85011	*Streptomyces scabrisporus* NF3	WGS	IIBiomedicas, UNAM	PRJNA377273	GCA_002024165	GCF_002024165	8	10958352	71,61	10491	9020	-	Yes	Aerobic	Plant
100226	*Streptomyces coelicolor* A3(2)	Complete		PRJNA242	GCA_000203835	AL645882, AL589148, AL645771	1	9054847	72	8325	8154	Branched filament	Yes	Aerobic	Multiple
1855352	*Streptomyces* sp. TLI_053	Complete		PRJEB16370	GCA_900105395	LT629775	1	9900053	73,63	8757	8065	-	-	Aerobic	-
227882	*Streptomyces avermitilis* MA-4680	Complete	ATCC 31267	PRJNA189	GCA_000009765	BA000030, AP005645	1	9119895	70,7	8106	7676	Branched filament	Yes	Aerobic	Multiple
260799	*Bacillus anthracis* Sterne	Complete		PRJNA236483	GCF_000832635	CP009541, CP009540	1	5409120	35,28	5854	5622	Bacilli	-	Aerobic	-
264445	*Streptomyces hygroscopicus* subsp. *limoneus* KCTC 1717	Complete	KCTC 1717	PRJNA302679	GCF_001447075	CP013219, CP013220	1	10537932	71,96	9983	8983	-	-	Aerobic	Terrestrial
452652	*Kitasatospora setae* KM-6054	Complete	ATCC 33774	PRJNA19951	GCA_000269985	AP010968	1	8783278	73,1	7709	7569		Yes	Aerobic	Terrestrial
455632	*Streptomyces griseus* subsp. *griseus* NBRC 13350	Complete		PRJNA20085	GCA_000010605	AP009493	1	8545929	72,2	7294	7136	Branched filament	Yes	Aerobic	Multiple
518634	*Bifidobacterium breve* DSM 20213	Complete		PRJDB57	GCF_001025175	AP012324	1	2269415	58,89	2016	1929	-	-	Anaerobic	-
54571	*Streptomyces venezuelae* ATCC 15439	Complete	ATCC 15439	PRJEB10583	GCF_001406115	LN881739	1	9034396	71,75	8487	8682	-	-	Aerobic	-
681645	*Corynebacterium pseudotuberculosis* C231	Complete		PRJNA40875	GCA_000144675	CP001829	1	2328208	52,19	2209	2053	Rod	No	Facultative	-

### Orthologous and orphan genes

**Fig 3:**
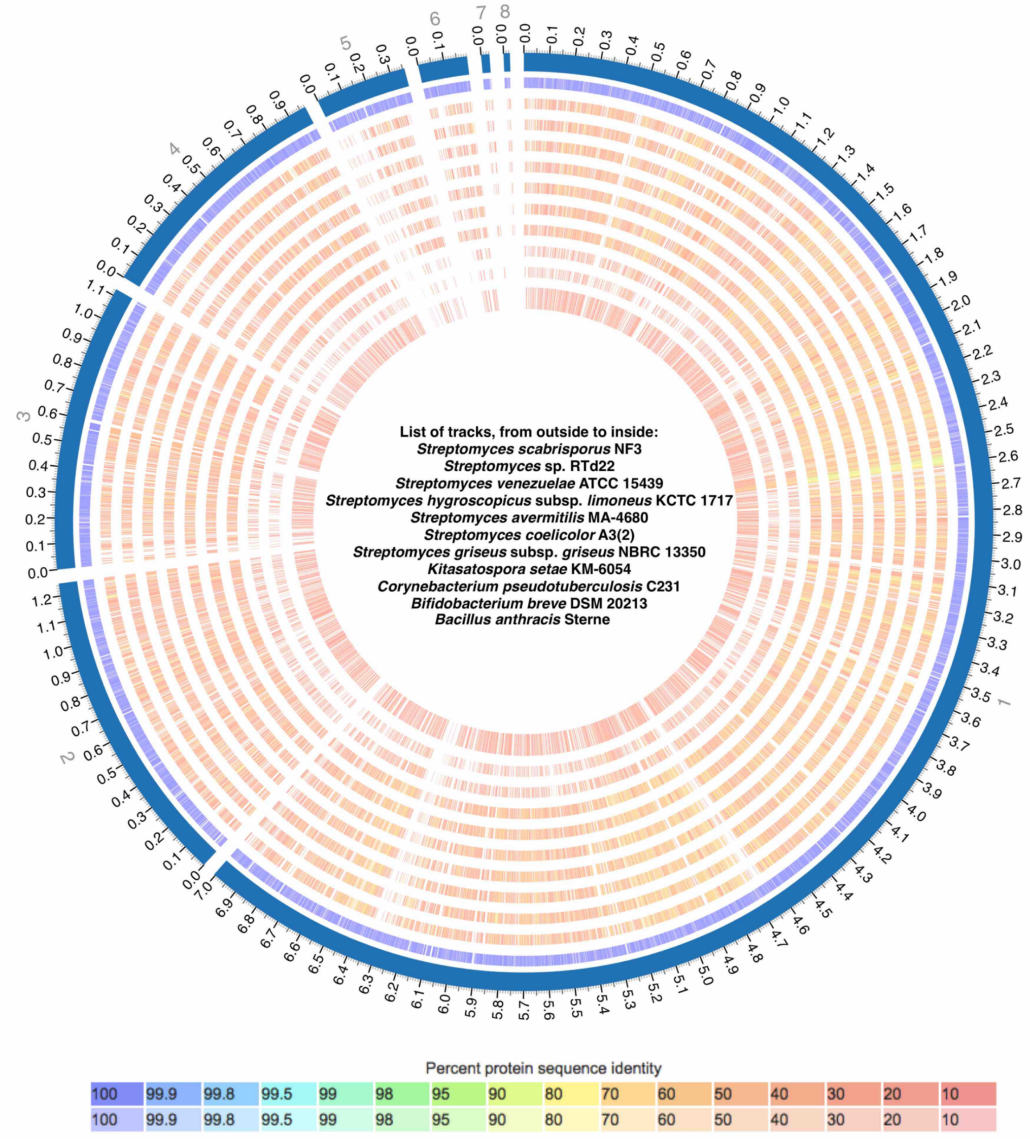
Proteome comparison between NF3 and the 10 complete genomes analyzed. The genomes are (from outside to inside): SS, SR, SV, SH, SA, SC, SG, KS, CP, BB, BA. The exterior solid line circle indicates the scaffolds of the reference genome–SS NF3. Each circle is comprised of lines, each of them indicating one protein homologous to a protein of the reference genome. An identity matrix indicating the relationship between the colors used and protein similarity is placed below the figure. The image was obtained from PATRIC utilizing the tool Proteome Comparison.

The proteomes of SS NF3 and of strains from 10 other different species (with complete genomes) with various taxonomic relatedness (Fig 2) were analyzed and compared using the PATRIC FIGFam software to identify orthologous gene families (OGF) (Fig 3, S1G Table). From the total of 5,784 OGF, only 421 families (7.3%) were shared by all 11 genomes (excluding single gene families). The unique OGFs from each genome ranged from 2 (*S*. *venezuelae* ATCC15439) to 266 (*B*. *anthracis* Sterne) (S2 Table). The genome with the most similar gene family profile to SS NF3 was *Streptomyces* sp. TLI_053, accounting for 1,842 common OGFs (Fig 3, S2 Table).

Compared to the other genomes, SS NF3 includes 166 unique genes whose orthologues were absent from all the comparative genomes used in this study; they were therefore selected as “orphan genes” (OG). Among these 166 OG, 129 belong to single-member gene families and 37 genes belong to multi-membered gene families (S1D Table). The second largest gene family for the OG (after hypotheticals) includes three glutathione S-transferase (GST) omega paralog genes. The GST omega subfamily is involved in cellular detoxification by catalyzing the GSH dependent reduction of protein disulfides, dehydroascorbate, and monomethylarsonate, activities which are more characteristic of glutaredoxins. Along with this GSTs, one iron-containing redox enzyme (DUF3050) are predicted to be involved in the reduction of oxidative stress. As the reduction of environmental oxidative stress is considered a factor positively contributing to plant growth[17,47,48], the presence of these unique genes could support strain SS NF3’s resistance to changing environmental conditions and indicate an endophytic lifestyle. These antioxidant genes are shared with SS DSM 41855. In SS NF3, these genes appear to be complementing the large array of xenobiotic degradation pathways present in the genome, such that NF3 could contribute to bioremediation of soils and water. Interestingly, among the NF3-specific orphan proteins, one unique sigma factor 24 (predicted to control adaptation to high temperatures in *E*. *coli*[49]), 2 peptidases and three luciferase genes (forming an operon) are present. Many of these unique genes belong to secondary metabolite operons as well as transporters (for instance one branched-chain amino acid ABC transporter). Alternative sigma factors activation was linked to reduction in the secretion of secondary metabolites in other *Streptomyces* sp.[50–53] and it is possible that its removal can allow SS NF3 to be used as a cell factory for its own or for heterologous metabolites production[49]. As no regulatory controller can be identified with the current data, remains to be investigated further if the luciferase operon is active and under which conditions.

The PFAM domains superfamilies that are overrepresented in the orphan genes are the P-loop containing nucleoside triphosphate hydrolase superfamily and amidohydrolase superfamily (S1C Table). These unique proteins may determine the host specificity of this organism, as the group encompasses detoxification enzymes (urease, creatinine amidohydrolase), antibiotic resistance proteins (beta-lactamase) (S1N Table) as well as cell wall modification enzymes (peptidoglycan N-acetylglucosamine deacetylases), all of which can contribute to plant interactions, as will be discussed further.

### Biosynthetic capabilities (KEGG metabolic mapping)

The endophyte’s functionality, as revealed by their gene content, is the result of their continuous and complex interactions with the host plant as well as other members of the host microbiome[16]. Understanding these functions at a molecular level and their participation in the global picture of plant-microbe interactions can be used to improve techniques of plant growth, biocontrol and bioremediation[54]. Microbiota research involving endophyte communities suggests high rates of metabolic activity related to carbohydrate and amino acid metabolism for these microorganisms[14]. Therefore, the biosynthetic capabilities of SS NF3 were inferred using metabolic mapping of its genome information onto the KEGG database.

#### Amino acid metabolism

Genomic analysis of the NF3 strain using the ModelSEED database (based on KEGG mapping) suggested prototrophy for all proteinogenic amino acids except pyrrolysine (S1F Table). From the non-proteinogenic amino acids, there are complete biosynthetic pathways for homocysteine, homoserine, hypotaurine, D-arginine, D-alanine, beta-alanine, D-glutamate, D-glutamine and ornithine. There is a high diversity of amino acid posttranslational modification genes, which means that they can produce extremely highly modified peptides, including D-amino acids, methylated amino acids and dehydrated amino acids and dozens of modified residues. The capacity to grow without needing external amino acids can be an advantage in poor nutrient environments and implies that the SS NF3 strain is likely to, at least in some segments of its life cycle, grow in soil communities; growth of another SS strain on various nitrogen sources was reported for peptone, yeast extract, (NH4)2SO4, NH4NO3 and L-asparagine[35].

Amino acids production is considered essential for establishing plant-bacteria interactions. Comparisons between endophytes and pathogenic strains of the same species indicate that endophytes would use a broader array of available amino acids and other acids as compared to the pathogens[55], suggesting a trend of endophyte prototrophy for amino acids. The biosynthetic capacity of a wide range of amino acids is also indicative of its advantage as an endophyte, since there are zones in plants which are poor in nutrients, becoming able to grow anywhere in the plant and providing nutrients to the microbiota existing there too. Bacterial protein secretion systems were previously successfully used for determining plant-bacterial interactions[1]. Transport systems in SS NF3 are dominated by genes involved in amino acid, peptide and protein transport (55% of transport genes are encoded by 92 genes), further supporting the idea that amino acids and peptides are essential for this bacteria’s interaction with its environment. An in-depth analysis of the specificities of these transporters by mutagenesis could provide further insights into the lifestyle of SS NF3.

#### Secondary metabolites

**Fig 4:**
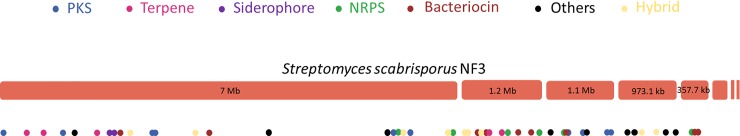
Localization of secondary metabolite clusters in *S*. *scabrisporus* NF3.

*In silico* genome analyses with the Secondary Metabolite Analysis Shell (antiSMASH) algorithm[56], BAGEL[57] and PRISM[58], and metabolic mapping using ModelSeed[59] on the SS NF3 genome revealed the presence of 413 genes in 45 clusters involved in the production of 59 different secondary metabolites, spanning polyketides (PKS–polyketide synthases), non-ribosomal peptides (NRPS–non-ribosomal peptide synthases and bacteriocins (Fig 4, S1K, S1L and S1P Table). The location of most clusters resides in the extremes of the larger scaffold and in the second largest scaffold, which is expected for *Streptomyces*, where most secondary metabolites clusters regularly reside at the extremities of the linear chromosome to prevent loss of central metabolism genes (Fig 4).

**Fig 5:**
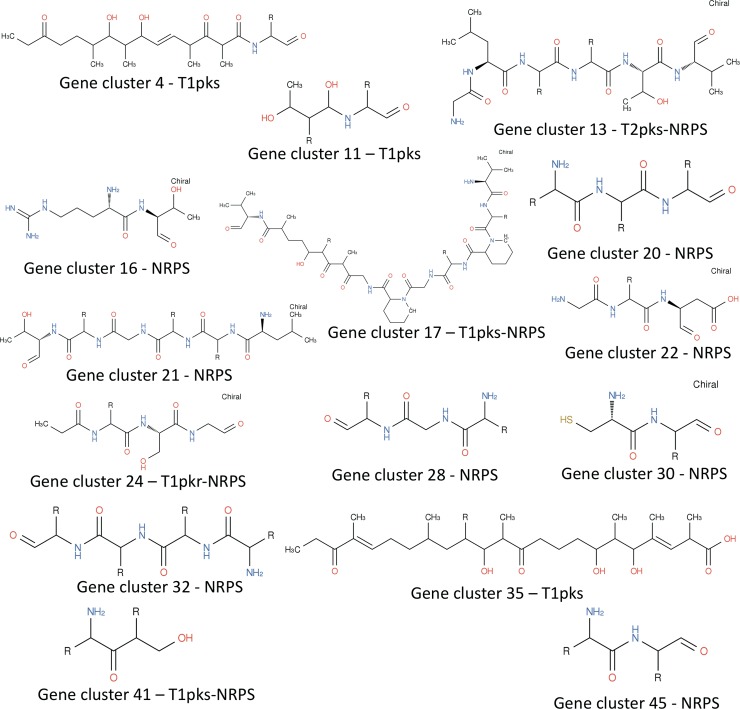
Predicted structures of secondary metabolites of *S*. *scabrisporus* NF3. The analyses were performed using AntiSMASH and PRISM.

A comparison between the abundance of secondary metabolite clusters of distinct types between the two SS strains reveals very similar amounts for the most abundant compounds, with higher numbers of NRPS and PKS for strain DSM 41855. Some clusters are shared with strain DSM 41855 and their products have previously been characterized, for instance alkylresorcinol, griseobactin, steffimycin and streptomycin. A high similarity (> 40%) with clusters previously characterized in other bacteria also exists for clusters potentially encoding spore-pigment, polyoxypeptin, piericidin A1 and hopene. From the remaining 38 clusters, 17 show relative similarity to known clusters (5% to 40%) and 20 are likely encoding completely new compounds. Structure prediction was possible using antiSMASH and PRISM for 15 compounds, among which 12 appear to be for molecules newly encoded in this genome (Fig 5).

#### Xenobiotic degradation

KEGG mapping of the main metabolic pathways for the genome of SS NF3 shows an enrichment of 3% in genes belonging to xenobiotic degradation pathways compared to the other genomes used in this study (data not shown, data from PATRIC pathways).

The xenobiotics predicted to be degraded by SS NF3 can be classified based on the strain’s capacity to use them for central metabolism. Directly feeding into the central metabolism are toluene, geranic acid, vanillate, benzene, nitrobenzene, phenol, bisphenol A, aniline, hydroquinone, salicylate, phenyl acetonitrile and phenylacetamide, while compounds that can be degraded to less toxic molecules, but do not supply energy for metabolism are urea, 6-mercaptopurine, fluorouracil, cyclohexanone, 1,1,1-Trichloro-2,2-bis (4-chlorophenyl)ethane (DDT), trichloroethylene, trinitrotoluene (TNT), trichloroethane, phenanthrene, pyrene, anthracene, 4-hydroxyphtalate, styrene, acrylamide, p-cymene and benzoic acid. Certainly, a closer view of the toluene and xylene degradation pathways in SS NF3 indicates the presence of more than one degradation route. The large diversity of genes involved in xenobiotic degradation and survival in toxic environments serves as an indicator of the extraordinary adaptability of SS NF3 and its elevated potential to be used in bioremediation or bioaugmentation of contaminated sites. In addition, these genes confer a general advantage as an endophyte, as it could help the host plant to degrade toxic compounds absorbed by the roots or feed off carbon sources which the plant does not utilize readily or produces in excess.

#### Carbohydrate-active enzymes (CAZymes)

CAZymes are enzyme families of structurally-related catalytic and carbohydrate-binding modules (or functional domains) of enzymes that degrade, modify, or create glycosidic bonds, like glycoside hydrolases, glycosyl transferases, polysaccharide lyases, carbohydrate esterases and some auxiliary redox enzymes such as polysaccharide mono-oxygenases[60]. In the context of plant interactions, various novel CAZymes with a role in degradation of plant polysaccharides could be identified by mining newly sequenced genomes or metagenomes[14,61–63]. Some studies go as far as using the presence of plant cell-wall degrading enzymes as a basis for bacterial classification as endophytes or as pathogens[14], under the hypothesis that endophytes are positively selected by the plant and only need to produce cellulases and pectinases, while pathogens requiring the degradation of the entire cell wall implement lignocellulose-degrading enzymes. In the SS NF3 genome, 1,071 genes classified in 130 CAZymes families contain carbohydrate-active domains (10.2% of the total gene count), among which the most abundant superfamilies are: histidine phosphatases (197 ORFs), Hsp70 chaperones (56 ORFs), dehydrogenases (50 ORFs), cellulose binding (29 ORFs), beta-lactamases (29 ORFs), response regulators (23 ORFs) and extracellular peptidases and solute-binding proteins (50 ORFs). Among the strain specific genes (76 CAZymes), 21 are predicted to be extracellular, cell wall or membrane attached, and are dominated by Hsp70 chaperones, solute-binding proteins and response regulators.

Root colonizing bacteria are sometimes equipped with enzymes enabling penetration of the root tissues such as lignocellulose or pectin degrading enzymes. The CAZymes involved in lignin degradation include lytic polysaccharide mono-oxygenases (LPMO) as well as auxiliary activities containing both ligninolytic enzymes and lytic polysaccharide mono-oxygenases. At least 29 unique genes from the SS NF3 genome belong to the AA families, while no enzymes of the LPMO type could be found (S1E Table). Using the FOLy database[64] to complement this data, we could establish that the SS NF3 genome contains two endo-1,4-beta-xylanases and 17 potential cellulases (S1I and S1J Table). This complex system of lignin and pectin degrading enzymes indicates a probable point of entry of SS, as a bacterium most commonly isolated from soil, through the plant root system, to reach other distant plant tissues.

#### Interference with plant metabolism

The close association between an endophyte and a living host is marked by the secretion of effector proteins that augment the host defenses and modify the host metabolism. The best-studied mechanisms of bacterial plant growth promotion include plants with resources/nutrients that they lack such as fixed nitrogen, iron, and phosphorus[65].

Nitrogen fixation is a metabolic property present in many plant-associated bacteria, especially the ones adapted to grow in the rhizosphere[1]. While no indication from the literature exists of *Streptomyces* strains able to fix nitrogen, these bacteria can participate in the formation and enhancement of root nodules[25, 66–68]. In SS NF3, the only nodulation gene we identified so far is *nolO* (PEG.9541), possibly mediating early stage recognition between bacteria and its host[69]. Despite not directly participating in nitrogen fixation, SS NF3 has all the genomic requirements to metabolize fixed nitrogen, through the urea pathway. The hydrolysis of urea is catalyzed by urease, yielding ammonium and carbon dioxide. The ammonium is then converted into glutamine by the glutamine synthetase encoded by the *glnA* gene. The bacterial urease is a multi-subunit protein complex, which consists of three structural subunits and several accessory proteins. We identified a cluster of genes coding for all subunits of the urease complex in the SS NF3 genome (PEG.1320 – 1322—urease, PEG.1318 –ureG, PEG.1319 - ureF). The genome also contains various copies of the *glnA* gene for glutamine synthetase (PEG.2332, PEG.4644, PEG.4669, PEG.6800, PEG.8087, PEG.8680), and a gene for a regulator of the GlnA proteins in response to the levels of nitrogen (PEG.4645).

In addition, second in abundance after amino acids and proteins transport genes, the genome contains a wealth of genes involved in the transport of nitrates/nitrites (PEG.426), iron (PEG.1363-1365; PEG.3259; PEG.5593; PEG.6834; PEG.8844; PEG.9278) and phosphorus (2 operons: PEG.1422 – 1429 and PEG.2798 – 2802). Among the iron transporters, there are some that transport siderophores bound to iron molecules. Diversity in siderophores of SS NF3 is discussed in the following section.

### Adaptive genomic changes specific to endophytes recognized in the SS NF3 genome

#### Symbiosis-related genes

Studies have revealed that beyond metabolism, other factors affect colonization and modulation of the bacteria—host interactions. Variation was observed in the distribution of essential genes related to hormone production and signaling, surface attachment, secretion, and transport between endophytes compared to pathogenic invasive bacteria [14, 65].

#### Virulence genes

Genes possibly involved in pathogen–host interactions (PHIs) were identified by their homologs in the PHI-base database [70]. About 7% (739 OGs) of SS NF3 genes had a hit in the PHI database, but only 2.5% (261 OGs) are extracellular or cell wall associated. Interproscan analysis of motifs present in the extracellular or cell wall associated virulence factors indicates the majority to be transporters and oxidoreductases (S1M Table), showing that SS NF3 acquired proteins could support symbiotic interspecies interactions.

#### Non-metabolic plant-endophyte interactions

In addition to metabolic collaboration, described in the previous paragraphs, endophytes use other strategies to modulate plant responses which encompass production of: a) compounds interfering with plant hormone signaling; b) antioxidants; c) chitin degradation molecules as a defense mechanism against fungi; d) siderophores for iron-storage; and e) antibacterial molecules such as bacteriocins to combat pathogens. Molecules belonging to these categories are abundant products of genes in the SS NF3 genome, as will be discussed below.

**Fig 6:**
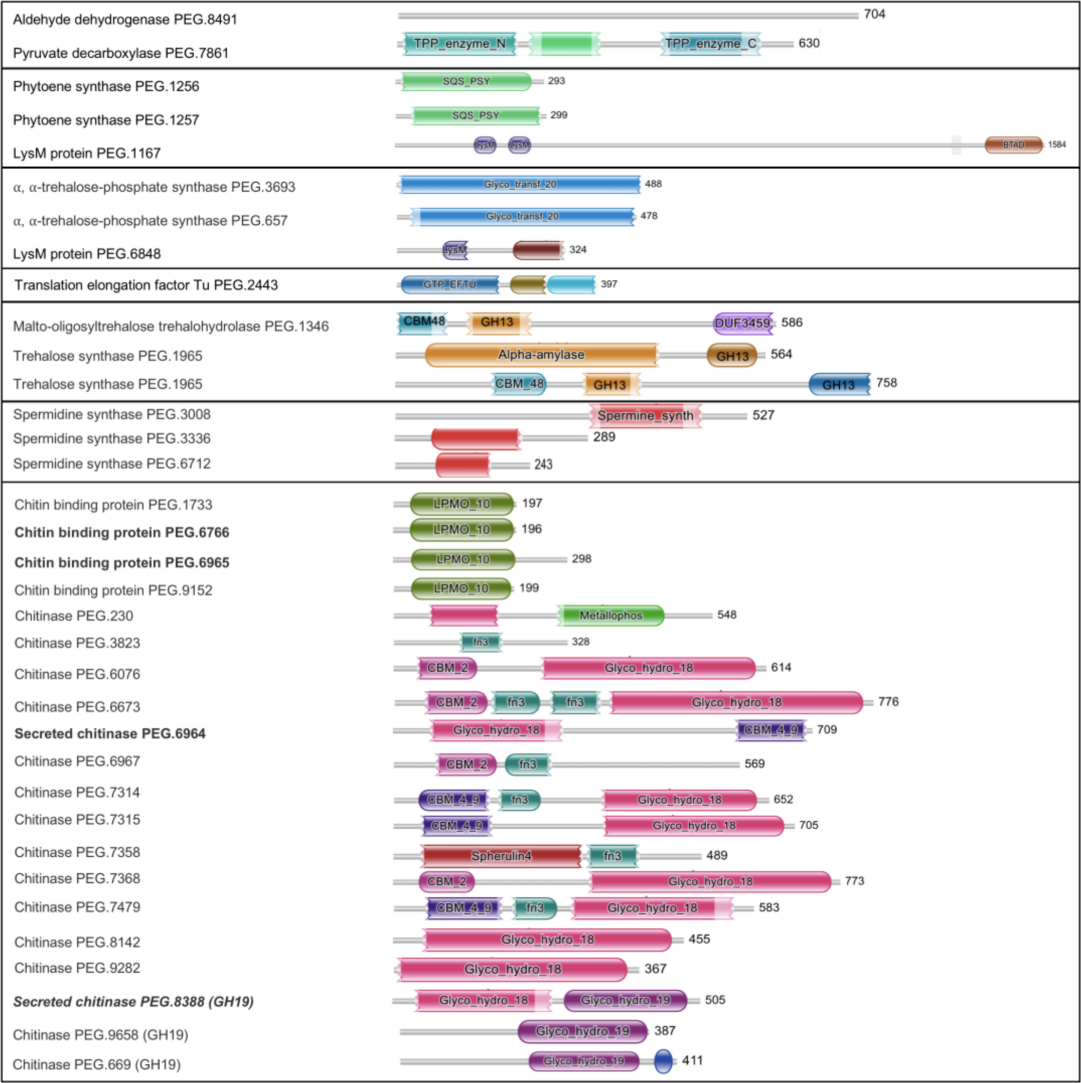
Domain composition for proteins from SS NF3 potentially involved in plant interactions. Legend: green–phytoene synthase; dark purple–LysM domain; dark blue–EF-Tu domain; pink–chitinase domain (GH18), red–spermidine synthase; orange–amylase. The analysis was performed using the HMMER scan tool from EBI. Only domains relevant for potential host interactions were annotated.

Plant hormones play key roles in plant growth and development and in the response of plants to their environment[13,71]. Microorganisms may also produce or modulate phytohormones under *in vitro* conditions[13,72]. Several microorganisms including endophytes can alter phytohormone levels and thereby affect the plant’s hormonal balance and its response to stress[25,73–74]. For instance, plant growth regulators (PGRs) such as auxins, cytokinins, gibberellins, strigolactones, brassinosteroids can be secreted by endophytes to enhance the host defense[75]. Soil or plant-associated microorganisms could produce IAA (indole-3-acetic acid) to promote plant growth[76–78]. In SS NF3, IAA can be produced from intermediates of the precursor tryptophan using the indole-3-pyruvic acid (IPyA) pathway, for which the genes are present in the genome (aldehyde dehydrogenase—PEG.8491 and indole-3-pyruvate decarboxylase—PEG.7861) (Fig 6).

Furthermore, in SS NF3, three individual genes and one operon could potentially modulate plant responses, by the synthesis and degradation of phytoene to carotenoid compounds. Carotenoids are natural pigments as well as lipid-soluble antioxidants[79]. In plants, carotenoids play important regulatory roles in optical system assembly, light harvesting and protection, photo-morphogenesis, non-photochemical quenching, lipid peroxidation, and seed dormancy and aging[79]. Phytoene synthase (PSY) is the first step in the biosynthesis pathway of carotenoids in plants, followed by phytoene dehydrogenase, an enzyme that converts phytoene into zeta-carotene. In SS NF3, an operon comprising the three genes coding for phytoene synthase, phytoene desaturase and squalene synthase is associated with the synthesis of carotenoids and hopanoids (Fig 6). Hopanoids act similarly to sterols in eukaryotes in that they condense lipid membranes and reduce permeability. In the case of prokaryotes, they provide stability under of high temperatures and extreme acidity due to the rigid ring structures[80,81]. Indeed, upregulation of squalene-hopene cyclase occurs in certain bacteria in the presence of hot or acidic environments[82]. In this case, carotenoids and hopanoids are likely to be shared with the host plant when conditions require it.

Rovenich et al. (2014) have recently discussed the possibility of ecological roles for plant effector proteins, especially those with the LysM/chitin-binding domain[83]. LysM domains are involved in non-covalently binding to the peptidoglycan of other bacteria[84]. Two genes with associated LysM domains in the genome of SS NF3 appear to be of interest to this discussion (Fig 6). One of them, PEG.1167, belongs to a protein of the tetratrico peptide repeat superfamily (TPR), which was implicated in plant hormone signaling in gibberellin, cytokinin and auxin responses as well as ethylene biosynthesis through direct interaction of its TPR domains with a 1-aminocyclopropane-1-carboxylate synthase isoform of plants[85]. The other lysM domain is associated with a cysteine peptidase Clan CA (PEG.6848). This class of enzymes is mainly used in plants to mobilize storage proteins in seeds. Protein bodies of seeds contain protease precursors. They are activated after germination and start degradation of the stored proteins[86]. Involvement of plant endophytes in seed development could explain instances in which association of plants with certain microbes lead to better crop yields[1,12].

Another type of signaling involves recognition by the plant immune system and induction of an elevated state of immunity that acts as prevention in the case of a pathogenic attack. EF-Tu is the most abundant bacterial protein that is recognized as MAMP by the receptor EFR in *Arabidopsis* sp. and other Brassicaceae family members[87]. One gene producing the translation elongation factor Tu (PEG.2443) is annotated in the SS NF3 genome (Fig 6), indicating that plant colonization by this Actinobacterium could further elevate plant resistance to pathogens.

Plant colonization by bacteria requires a high tolerance for environmental stresses, especially osmotic stress, and production of trehalose as one of the main factors contributing to such resistance[73]. Trehalose of bacterial origin was among the most strongly induced metabolites produced following inoculation of soybean (*Glycine max*) root hairs by the nitrogen-fixing symbiotic bacterium *Bradyrhizobium japonicum*[73], functioning as an inducer of the endophytic relationship. In the genome of SS NF3, two operons for trehalose synthases, two independent synthases (PEG.3693 and PEG.657) and two degradation genes are contributing to trehalose metabolism. The first operon is formed by a malto-oligosyl-trehalose-synthase and a malto-oligosyl-trehalose trehalohydrolase (PEG.1346-1347). The second operon (PEG.1965 – 1967) codes for a protein with an alpha amylase with a malt amylase domain, one phosphotransferase and two trehalose synthases. The presence of seven genes involved in at least three different pathways for trehalose production denotes a high dependence of the bacteria for this carbohydrate and its probable importance in SS NF3’s lifestyle. Other synthase complexes involved in bacterial stress responses with many homologs in SS NF3 (PEG.3008, PEG.3336 and PEG.6712) are spermidine synthases. Spermidine derivates contribute to tolerance to drought and salinity in plants.

**Fig 7:**
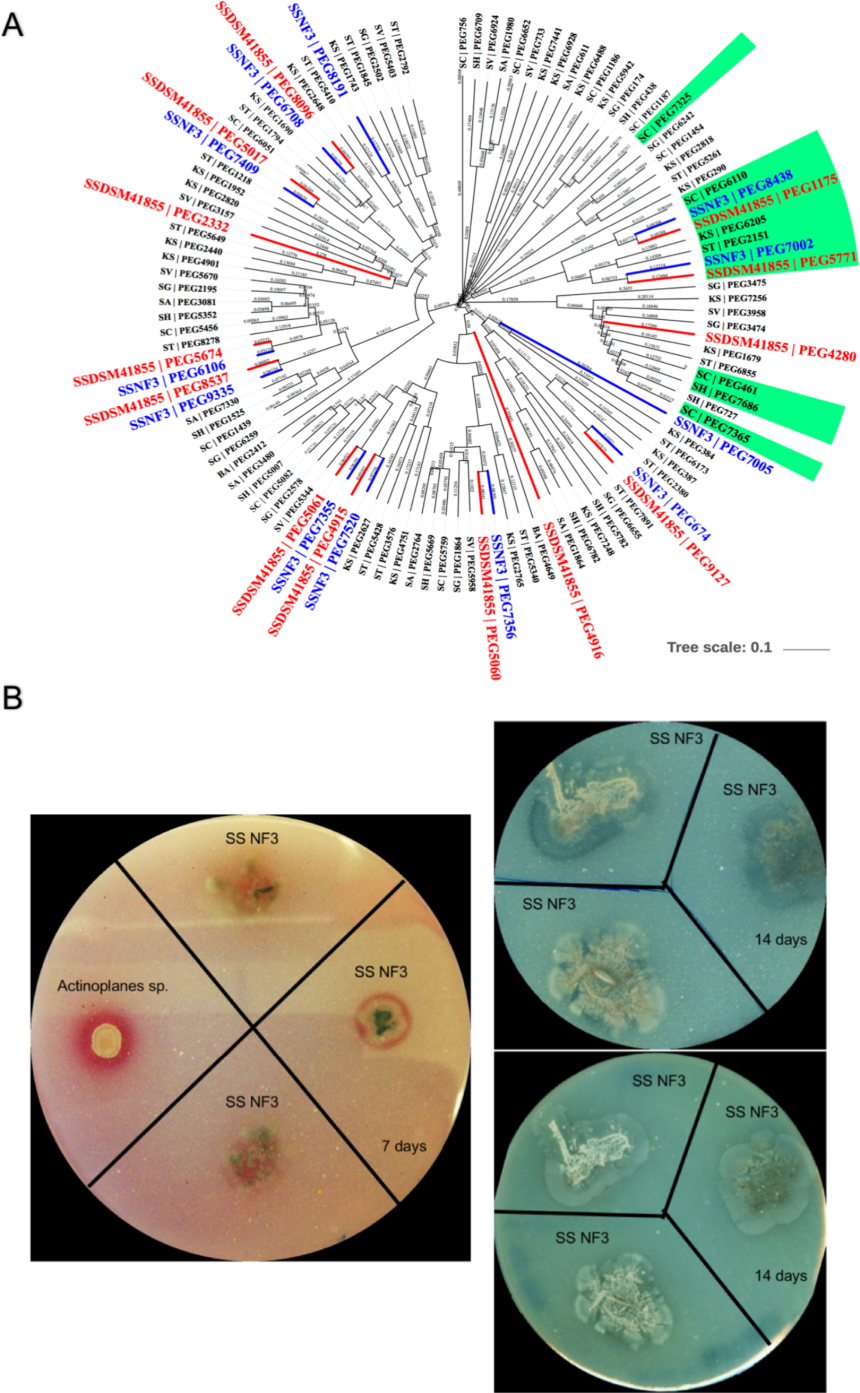
Chitinase genes and chitinolytic activity. A. NJ tree of all chitinase genes in the compared genomes. The MSA was produced using MUSCLE on all 116 chitinase sequences. Branches from proteins belonging to genomes S. scabrisporus NF3 and DSM 41855, as well as all secreted chitinases, are color labeled, respectively: blue, red and green. B. Expression of chitin degrading genes in SS NF3. Left side—chitin degradation at 7 days after inoculation. Right side upper level—chitin degradation at 14 days after inoculation. Ride side lower level—spore formation after growth on chitin for 14 days.

Another essential mechanism for bacteria to become useful partners in their interactions with plants is through fighting off pathogenic attacks. Siderophores, bacteriocins and antibiotics are three of the most effective and well-known mechanisms that an antagonist can employ to minimize or prevent phytopathogenic proliferation[88]. In addition, chitinases target fungal cell walls to release chitin fragments that activate immune receptors, leading to further chitinase accumulation to induce hyphal lysis. Chitinases are enzymes that catalyze the hydrolysis of the beta-1,4-N-acetyl-D-glucosamine linkages in chitin polymers present in the cell wall of microorganisms [89]. They are produced mostly in filamentous fungi, which are known to produce up to 20 different chitinases. *Serratia marcescens* is one of the bacteria with the highest reported chitinolytic activity, counting five different chitinases[90–92]. The SS NF3 genome contains 14 chitinases belonging to the glycoside hydrolase (GH) 18 family and 4 chitinases from the GH19 family (S1O Table, Fig 6). A BLAST analysis shows that these chitinases are unique for the species SS but shared with the other sequenced strain of the species: DSM 41855. Eight of them have a carbohydrate binding domain. Among them, four genes (PEG.6964-6968) form a cluster containing one secreted chitinase with a degradation domain (Fig 6), two chitin-binding proteins and an esterase. Exceptionally, the SS NF3 genome also contains four chitin-binding proteins, with a total of 20 chitin degradation or binding genes (Fig 6, S1O Table). In addition, a NJ tree based on a MSA produced using MUSCLE containing all the chitinase genes from the compared genomes (116 sequences) (Fig 7A) indicates that the SS NF3 chitinases are not paralogs, encompassing a large structural variation and belonging to different branches of the tree.

This raises the question of what the necessity could be for this high number of various chitinases. The foreseen explanation is that they could be involved in the biological control against particular fungal or nematode plant diseases[88,93]. In support of this hypothesis, recent research evaluating the chitinase activity of 63 different strains of Actinobacteria isolated from soil samples of China found that the strain identified as SS Bn035 displayed the best chitinase activity, indicating that chitin degradation could be a species characteristic. The antifungal activity of strain Bn035 was tested against four pathogenic fungi (*Bipolaris sorokiniana*, *Fusarium oxysporum*, *Rhizoctonia solani* and *Pythium capsici)*, involved in turfgrass root rot disease, with highly positive results[35]. The chitinolytic activity of SS NF3 in laboratory conditions was assayed in solid plates containing colloidal chitin as a sole carbon source. We could observe that the bacteria present chitinolytic activity after 7 days of incubation and supports the formation of spores after 14 days of incubation (Fig 7B).

**Fig 8:**
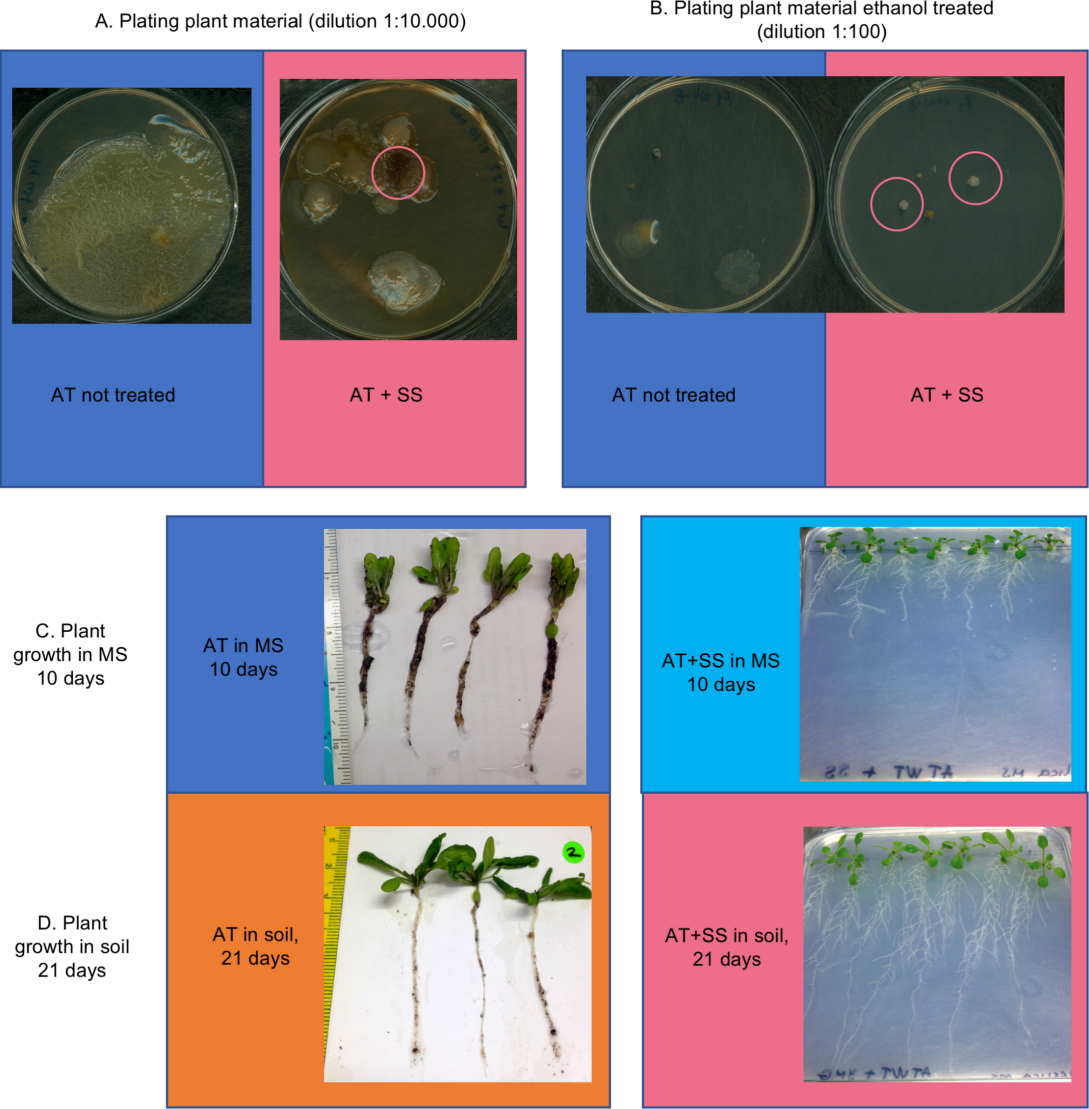
Colonization by SS NF3 of the plant *A*. *thaliana* (AT). Pink circles are used to indicate presence of SS NF3 colonies, which display a characteristic crumpled surface and production of red/orange secondary metabolites. A. Dilutions of plant material after 10 and 21 days of growth of plants inoculated with the bacteria (dilution 1:10.000). B. Dilutions of the same plant material after ethanol treatment for 90 seconds (dilution 1:100). C. Plant growth after 10 days in MS plates. Left, control plants with uninoculated plants. Right, plants whose seeds were inoculated with SS NF3. D. Plant growth after 21 days in regular soil. Left, control plants with uninoculated plants. Right, plants whose seeds were inoculated with SS NF3. (data shown is representative for 2 independent experiments).

Many bacteria closely interacting with plants produce secondary metabolites as agents needed for nutrient uptake (Fig 8). For instance, iron bioavailability is reduced in aerobic environments, including soil. To overcome this limitation, microorganisms have developed different strategies, such as iron chelation by siderophores. Bacteria producing siderophores have the ability to suppress phytopathogens and could be of significant agronomic importance[18]. Ten siderophore synthethase clusters containing 19 siderophore synthetases potentially involved in iron acquisition were found in the SS NF3 genome, as well as various iron-siderophore transporters (S1P Table).

After mining the diversity and abundance of the genes present in SS NF3 in comparison to the group of 10 other complete genomes and DSM 41855, we conclude that overall, this organism has a larger potential for host interaction, secondary metabolites production and protein degradation (S1P Table). The sum of all genes included in PFAM families related to the production of secondary metabolites is highest in the strain NF3 (87 families) compared to all the other genomes (<75 families), indicating a significant enrichment for these genes (by 16%). The ability of SS NF3 to express this remarkable genomic potential remains to be investigated by proteomics/metabolomics, in different growth conditions, in future studies.

### Colonization of the plant *Arabidopsis thaliana* by SS NF3

Genome mining of the SS NF3 genome as well as its isolation source seems to indicate a potential for plant colonization, hypothesis which we tested under laboratory conditions by inoculating plants of *A*. *thaliana* (AT) with the bacteria and comparing with an untreated plant group. After a full growth cycle (21 days), plants initially treated with SS NF3 continued to carry the bacteria, despite it needing to compete with the soil microbiota and plant defense systems (Fig 8A). In addition, dilutions of plant material after a 90 seconds ethanol treatment, plated on YMG, showed (for some plants uniquely) colonies of SS NF3 (Fig 8B). Control experiments using only mycelia of NF3 treated with ethanol for 90 seconds did not result in colonies (data not shown), indicating that the bacteria were residing at the interior of the plants and not on their surface. Plants treated with SS NF3 showed a different phenotype in both MS plates (at 10 days) but not after the period of growth in the soil. The observed changes in plant physiology included visibly smaller plants with shorter roots and a reduction in lateral roots formation for growth in MS plates (Fig 8C and 8D). The changes in plant physiology in MS plates could be due to a competition for nutrients between the bacteria and the plants, since colonies of SS NF3 are clearly visible at the inoculation site. This effect is not maintained when plants are transplanted in soil, where, in the presence of full soil microbiota and despite its presence at the end of the experiment, SS NF3 does not appear to have an effect on plant physiology. This a preliminary conclusion and further analysis of soil microbiota changes, production of plant hormones by plants and bacteria, and discrete changes in the plant need to be investigated. Another explanation for the data is that AT is not the most common host/ type of host for SS NF3 and larger physiological effects could be observed when changing the plant model.

**Fig 9:**
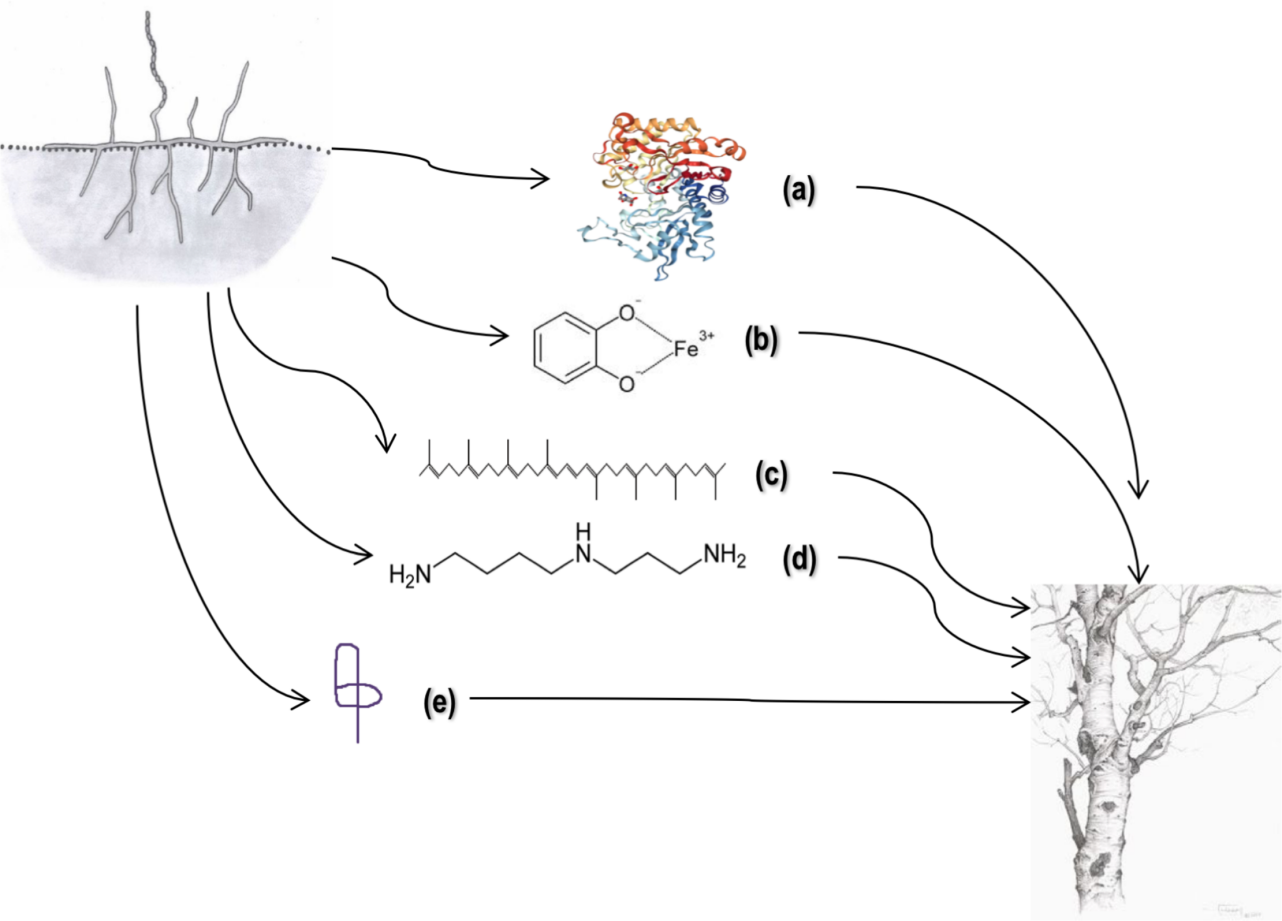
Genes involved in symbiotic interactions between SS NF3 and *A*. *adstringens*. (a) Chitinase family 18 (PDB 4Q22), (b) Siderophore, (c) Phytoene, (d) Spermidine, (e) Bacteriocins (here a lassopeptide).

In summary, the SS NF3 genome contains a diverse range of genes encoding for metabolites potentially important for this strain’s adaptation to an endophytic lifestyle (Fig 9), and investigation of its singular activities could provide a platform for understanding mechanisms of plant—microbe cross-talk.

## Conclusions

In this study, we investigated the genome of the endophytic SS NF3 strain, which presents remarkable biotechnological capabilities, with the interest to reveal its functional attributes in microbe-plant interactions. Proteome comparison with other taxonomically related as well as distant bacteria revealed an enrichment in metabolic pathways related to amino acid and protein synthesis and carbohydrate degradation as well as the presence of more than 50 clusters encoding potentially active compounds against plant pathogens (S1F Table). Metabolic mapping revealed prototrophy for 28 amino acids, nitrogen fixation capacity through the urea pathway and the potential for synthesis of hormones able to modulate plant growth (Fig 8). Experimental work confirmed the hypotheses that resulted from genome mining, such as the potential for plant colonization and chitin degradation.

Further research into the unique gene pool of this species with plant growth potential and a diversity of xenobiotic degradation and chitinolytic enzymes could provide valuable ways to improve agricultural practices by use of plant probiotics and decreasing the use and persistence of chemicals currently used for plant growth.

## Materials and methods

### Strains, media and culture conditions

The strain SS NF3 is deposited and available from the Institute of Biomedical Research Culture Collection, UNAM. The bacteria were grown in YMG (4 g/L yeast extract [Difco], 10 g/L malt extract [Difco], 4 g/L dextrose) in liquid cultures and incubated at 26°C for mycelia production and in YMG with 15 g/L agar [Difco] for growth on solid plates; cryogenic stocks were stored YMG with 15% glycerol at -80°C.

The *Arabidopsis thaliana* plants used are Columbia-0 ecotype. Seedlings were grown on vertical plates with 0·2× Murashige Skoog salts (MP Biomedicals) and 1% sucrose, as previously described[94].

### Genome and proteome comparisons

The closest taxonomic relative of *S*. *scabrisporus* NF3 with an annotated genome is *S*. *scabrisporus* DSM 41855, with which it shares approximately 98% of predicted coding sequences (CDSs). Due to the inadequate quality of the genome sequence for the latter, it could not be included in many of the genome analyses. A bi-directional BLAST analysis using the two *S*. *scabrisporus* genomes was performed using PATRIC’s Proteome comparison tool. The strains used for proteome comparisons with PATRIC’s Proteome comparison tool as well as orthologous group families comparison using FIGFams are: *Streptomyces coelicolor* A3 (2), *Streptomyces* sp. TLI_053, *Streptomyces avermitilis* MA-4680, *Streptomyces hygroscopicus* subsp. *limoneus* KCTC 1717, *Kitasatospora setae* KM-6054, *Streptomyces griseus* subsp. *griseus* NBRC 13350, *Streptomyces venezuelae* ATCC 15439, *Corynebacterium pseudotuberculosis* C231, *Bacillus anthracis* Sterne and *Bifidobacterium breve* DSM 20213 = JCM 1192. Comparison of the gene families was performed in PATRIC using a Pearson pairwise average linkage correlation.

Other genomic comparisons and data analysis were performed using the online tools BLAST at the NCBI and Sanger web sites (www.ncbi.nlm.nih.gov/BLAST/ and http://www.sanger.ac.uk/), the Kyoto Encyclopedia of Genes and Genomes (www.genome.jp/kegg/), and ModelSEED (http://modelseed.org/) for metabolic pathway analysis.

For the enrichment analysis for genes potentially involved in host interaction, the PFAM family search was performed for family annotations including: polyketide, non-ribosomal, bacteriocin, lantipeptide, spermidine, indole, phytoene, chitin, chitinase, siderophore, choosing families with more than 2 members and more than 3 genomes. The results of the bacteriocin presence in the first column were obtained with Bagel3 (S1P Table).

### PFAM domain annotation

InterProScan 5 was used to annotate the Pfam domains in the *S*. *scabrisporus* NF3 and comparative genomes. An enrichment test was performed using Fisher’s exact test embedded in the Blast2Go module. Secreted proteins were predicted using PSORTb and SignalP[95] (S1A Table).

### Secondary metabolites operons detection

Sequence analysis was performed by combining antiSMASH version 3.0.4[56], BAGEL3[57] and PRISM [58] genomic analysis platforms.

### Phylogenetic analyses

In order to evaluate the taxonomical relatedness among the compared organisms, we used a multiple sequence alignment built with Kalign[46] of 4 concatenated protein sequences from each organism: 16S rRNA, gyrB, rpoB and recG. These proteins were chosen based on previous reports of phylogenomic analyses in prokaryotes according to their presence in all selected species, absence of additional fused domains, no subjection to Horizontal Gene Transfer (HGT), and completeness. The tree was built by calculating pairwise distances using PHYLIP Neighbor Joining with a Kimura distance matrix model in UGENE[96].

The relatedness of all chitinase genes from all compared genomes was evaluated using a multiple sequence alignment built with MUSCLE[97], and visualized by building an unrooted tree by calculating pairwise distances using PHYLIP Neighbor Joining[98] with a Kimura distance matrix model in UGENE. Branches from proteins belonging to genomes *S*. *scabrisporus* NF953 and DSM 41855, as well as secreted chitinases, were color labeled, respectively: blue, red and green.

### Chitin degradation assay

Chitinolytic activity was determined as described[99]. Agar plates (2%) supplemented with colloidal chitin at 0.5% (SIGMA), partly hydrolyzed by stirring in 0.5 M HCl for 2h, were inoculated with strain SS NF3 and incubated at 28°C. Hydrolysis zones were detected as cleared zones after 7 and 14 days of incubation.

### Colonization of *Arabidopsis thaliana*

Seeds of Arabidopsis *thaliana* Col-0 used in this study were disinfected in 20% sodium hypochlorite and 0.01% of Tween-20 for 15 min. Then seeds were stratified at 4°C for 4 days under dark conditions and sown on square petri dishes containing 0.2X Murashige and Skoog salts (MS) (MP Biomedicals), 0.05% MES (SigmaAldrich), 1% sucrose (SigmaAldrich) and 1% agar (Becton, Dickinson and Company) at pH = 5.6. At the moment of inoculation in MS plates, one group of seeds were inoculated with 10^7 CFU of bacterial mycelium previously grown in YMG for three days and washed (3x) in phosphate saline buffer (PBS). Bacteria was used as mycelia since spores could have survived in MS and soil and confounded the experiment. The plates were vertically incubated in a growth chamber at 22°C under long-day (LD; 16 h light/8 h dark) conditions with a light intensity of 110 μEm-2 s-1 for 4 days. Plants were transplanted at day 10 in complete unsterilized soil (3 plants/pot) and grown for an additional 11 days. After a complete 21 days of growth, plants were harvested, homogenized and a set of serial dilutions between 100 and 10000x was plated on YMG. One set of inoculated plants were pretreated with ethanol for 90 seconds before homogenization with the purpose of eliminating plant surface microbiota.

## Supporting information

S1 Fig Position of the genome size of *S*. *scabrisporus* NF3 within the genome length distribution of sequenced *Streptomyces* isolates. All *Streptomyces* genomes from the PATRIC database with more than 30x coverage and a size larger than 6Mb (the smallest reported *Streptomyces* has 6.8Mb [99]) were included in the analysis.
(TIF)

S1 Table Supplementary analyses performed on the analyzed genomes. a. PATRIC annotation data and supplementary analyses summary: PSORTb, SignalP, PHI hit (virulence), PFAM hmm domain, PFAM superfamily, CAZymes families BESC (PFAM based), CAZymes annotation BESC (PFAM based), CAZymes families dbCAN (HMM based), CAZymes description.
b. RAST annotations for the *Streptomyces scabrisporus* NF3 genome.
c. PFAM annotations for the *Streptomyces scabrisporus* NF3 genome.
d. Unique gene families (FIGfam) for the *Streptomyces scabrisporus* NF3 genome.
e. Secreted unique proteins for *Streptomyces scabrisporus* NF3 as resulting from the comparative proteomes analysis.
f. KEGG mapping for secondary metabolites pathways in *Streptomyces scabrisporus* NF3.
g. Proteome comparisons for *Streptomyces scabrisporus* strains NF3 and DSM 41855.
h. All selected genomes, determination of OG.
i. CAZymes classification of genes of *Streptomyces scabrisporus* NF3.
j. CAZymes unique for the *Streptomyces scabrisporus* NF3 genome.
k. Detection of secondary metabolite clusters using automated engines: AntiSMASH results.
l. Detection of secondary metabolite clusters using automated engines: BAGEL3 results.
m. Positive BLAST results for genes from the *Streptomyces scabrisporus* NF3 genome in the PHI virulence database.
n. Antibiotic resistance genes recognized in the *Streptomyces scabrisporus* NF3 genome.
o. Genes annotated as chitinases in the *Streptomyces scabrisporus* NF3 genome.
p. Enrichment analysis for genes belonging to PFAM families with annotations including: polyketide, non-ribosomal, bacteriocin, lantipeptide, spermidine, indole, phytoene, chitin, chitinase, siderophore, choosing families with more than 2 members and more than 3 genomes. The results of the bacteriocin presence in the first column were obtained with Bagel3.
(XLSX)

S2 Table Orthologous gene families mapping for all compared genomes. (XLSX)
