# Supplementary figures and images for: Genome mining of Streptomyces scabrisporus NF3 reveals symbiotic features including genes related to plant interactions
Source: PLoS One. 2018 Feb 15;13(2):e0192618. doi: 10.1371/journal.pone.0192618 (PMC5813959; doi:10.1371/journal.pone.0192618)

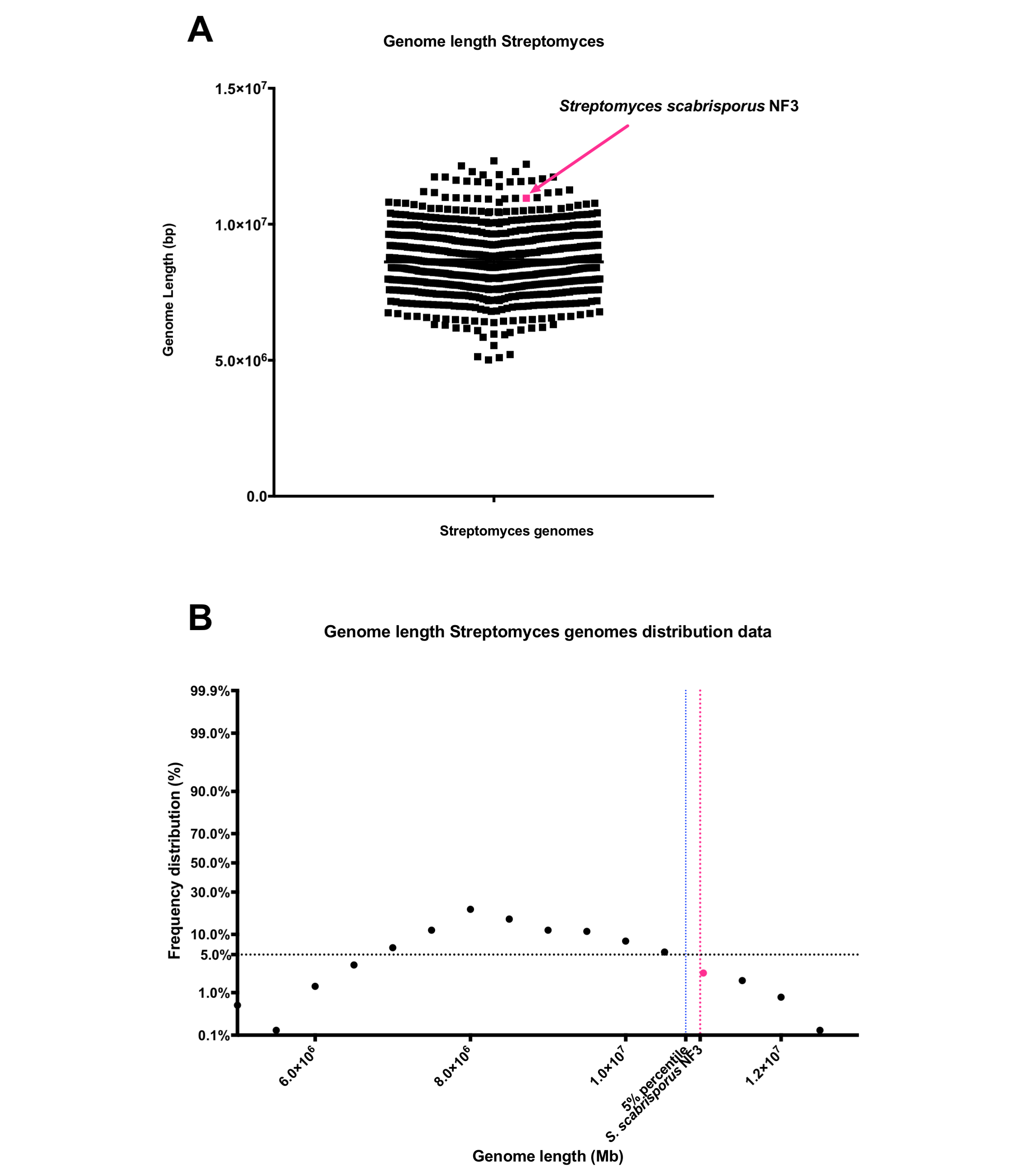

Supplement: S1 Fig — All Streptomyces genomes from the PATRIC database with more than 30x coverage and a size larger than 6Mb (the smallest reported Streptomyces has 6.8Mb [99]) were included in the analysis. (TIF) [file pone.0192618.s001.tif]
